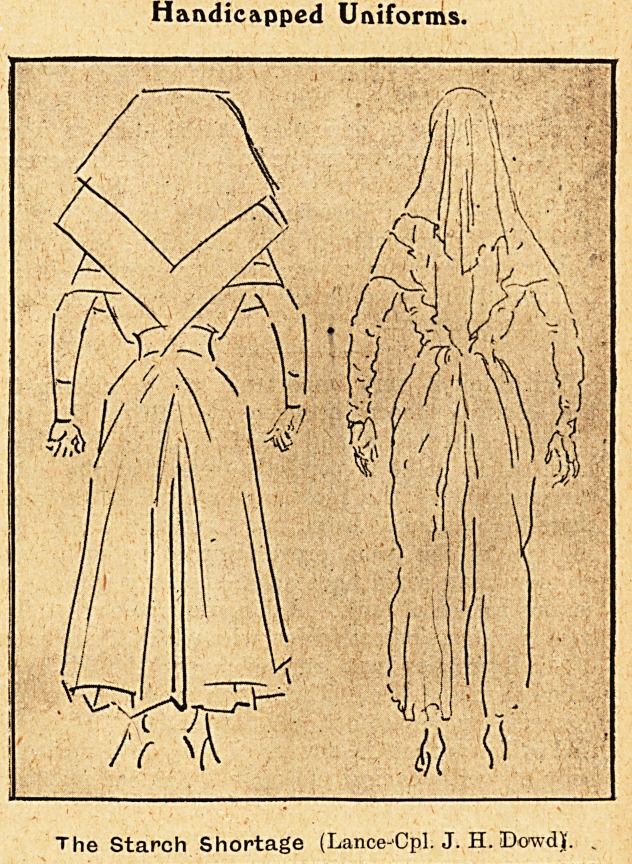# Round the Hospitals

**Published:** 1917-12-15

**Authors:** 


### ROUND THE HOSPITALS.

We have received a further correspondence which has taken place between the secretaries of the Royal British Nurses' Association and the College of Nursing, Limited. We have not room to insert it, nor is this necessary, seeing that we have already published, with one exception, the material facts in the various letters which have already appeared in our columns. The exception is a letter from the Honorary Medical Secretary of the Royal British Nurses' Association to the Clerk of the Privy Council, in which he sends a copy of the resolutions of its Council passed on September 27 last, in which it was decided not to accept the alterations suggested by the Privy Council—*i.e.* in effect to withdraw the application for an amended Charter. The Honorary Secretary thanked the Lords of the Council for their courtesy, and apologised for the delay in the reply to the Privy Council's letter of August 1, 1917. Sir Almeric Fitzroy, Clerk to the Council, in his reply merely stated that the decision arrived at had been duly noted by the Lords of the Council.

It is a matter of importance, however, to make clear the grounds on which sixteen nurses in a small meeting of the Council (the honorary officers and two members present not voting) refused to accept the Privy Council's alterations in the draft of the amended Charter. To prevent any misunderstanding we reproduce the points, in practically the identical words spoken by Sir James Crichton Browne, a vice-president of the R.B.N.A., as chairman of the Council meeting on July 5, 1917. Clause D in the Supplemental Charter ran as follows:—"The making and maintaining of an official register of persons qualified to act as nurses." The Privy Council previously struck out the words "the official register" and substituted "a Roll." (In the old Charter the words are "a list" and confer no right to a register.) Sir James proceeded:—"The word 'Roll' was suggested to the Council by the Roll of Midwives, and I think, owing to the action of your Honorary Secretary and Mr. Stanley, the Privy Council have given way upon that point, and have agreed that the clause shall run as follows:—'The making and maintaining of a register of persons qualified to act as nurses.' It seems to me that the retention of the word 'Register' instead of the word 'Roll' and without the prefix 'official' in Section 2, Clause D, meets the legitimate requirements of the Corporation. The Register, which it would be the purpose of the Corporation to make and maintain, would be 'official' so far as the Association is concerned, and if we did not obtain the Supplemental Charter we should not be entitled to use the word 'register,' but must fall back on the word 'list,' which has no official signification. I think it is a great point gained that the Privy Council gave way on this."

Sir J. Crichton Browne next dealt with Clause E of Section 2 of the amended Charter, the first two lines of which read:—"To promote legislation to provide for the State recognition of and protection of the official Register." The Privy Council suggested that the word "an" should be substituted for "the" before "official Register." They proposed that a register of one kind or another should be kept. "Now I understand that it has been suggested that this Supplemental Charter is an attempt to forestall Parliament. As regards that I would observe that the first line of Clause E, Section 2, 'to promote legislation' etc., cannot be construed into an attempt to forestall the action of Parliament, and should legislation for the official or statutory registration of nurses be undertaken it will be for Parliament to define what that register shall be, and the qualifications of those whose names shall be inscribed upon it, but it will be within the competency of any Association or any individual to promote the recognition and protection of any existing Register. If, however, it be that the word "official" is objected to, as liable to be understood to imply some existing claim to statutory recognition, then the substitution for the words 'the official register' of the words 'the Register of the Corporation' should, I think, meet the views of the Privy Council. Of course, if the Privy Council agree that we are to have a Register, they admit that it becomes a Register not merely of the British Nurses' Association, but of the combined body, and I think that is a strong position. They left the clause in a much more favourable form than I expected. If they accept the words:—'The State recognition and protection of the Register of the Corporation,' this will give you—*i.e.* the R.B.N.A.—a status against any other register; it will practically grant you protection for the Register of the Corporation." It will thus be seen that Sir J. Crichton Browne made it perfectly clear (the Privy Council being willing to adopt the words or similar words to those stated by Sir J. Crichton Browne) that the amended Charter gave the Royal British Nurses' Association and the College everything in regard to registration which the Privy Council had it in their power to grant.

At first sight it was fair to assume that the sixteen nurse objectors could not have understood this, or they would not have wished to break the agreement with the College. It appears, however, from the *Nurses' Journal* of November that, according to Dr. Stewart, "many nurses were misled by the circular of the College," and he felt it would not do to consent to the amended Charter while nurses still had this idea. Sir J. Crichton Browne replied: "Unfortunately that appears to have been so," but if the R.B.N.A. carry out their amalgamation with the College they "will have 4,000 members of the British Nurses' Association and 5,000 of the College of Nursing—9,000 nurses. It is certain that with that number on the Register it will be accepted as an accomplished fact by the State. It will become the basis of the State Register." But Mrs. Latter

**ROUND THE HOSPITALS**—(*continued*).

maintained that they "ought to have some sort of assurance. We must be certain of our position in the matter." It was pointed out that the College did not promise that their nurses would be on the State Register. They could not promise it. They only promised to draft a Bill, and any sensible woman reading that would understand that until the Bill was passed they could not have a State Register. Sir J. Crichton Browne added: "I think it is certain that the Register would be accepted."

Mrs Latter was not, however, satisfied. She required "powers to fulfil all pledges," *i.e.*, to have the words "official Register" in Section 2, Clause D, the words which it was beyond the power of the Privy Council to pass. In the course of subsequent discussion Sir J. Crichton Browne stated: "The Privy Council evidently think the word 'official' may be understood to mean 'statutory.' The word 'official' is used a great deal in Government offices—a letter is called 'official.' It is a mere indication that it has formal recognition. I think it is a great pity to risk the amalgamation breaking down for a mere quibble. I am of the opinion that the Privy Council will not give way on this small point. It is only formal whether it stands as 'the Register of the Corporation' or 'the official Register.' I think it is very satisfactory that the Privy Council gave up the word 'Roll' and adopted the word 'Register.'" Mrs. Latter and Mr. Paterson refused, however, to be satisfied. Mr. Paterson maintaining that the College had given their pledge that the nurses would be on the State Register. (This was clearly an error on his part, as the College Bill demonstrated.) Sir J. Crichton Browne declared: "No such promise could be given by any public body, but there is every probability that this will be fulfilled. Parliament could not ignore a Register of 9,000 nurses." Still Mrs. Latter persisted in her objection, and Mrs. Latter and her supporters had their way. Sir J. Crichton Browne confessed he was "disappointed, very disappointed."

We think it very important, on the closing of the correspondence between the Royal British Nurses' Association and the College of Nursing, Limited, to make it perfectly clear that the non-carrying out of the agreement arrived at between these institutions is based upon grounds which, on the face of the facts, prove them to be such that no mere man could have acted similarly without a serious breach of faith. Again, we lament that the good intentions on both sides, with which the scheme of amalgamation was entered upon, should have failed to materialise. Those who read the *Nurses' Journal* for November and December 1917 will find evidence that the sowers of tares have misled a body of well-meaning and possibly, as one member put it, quite ordinary people. The *bona-fides* of the majority at any rate is unquestionable, but more than one member declared, in effect, that their aim was an official Register to get State Registration. And yet the voting of the sixteen nurses to tear up the agreement with the College was the most certain course, each could take, to cause the maximum delay they could bring about in the granting of State Registration. The pity of it too, seeing that during the months of delay caused by the R.B.N.A. indecision and misunderstanding, the College Register has grown by leaps and bounds. It now approaches, we understand, 8,000 members, so that the nurses on the Register of the College and the R.B.N.A. combined, would to-day exceed the number

**A Ward (Muir) Orderly** (Lance-Cpl. J. H. Dowd).

necessary, in Sir J. Crichton Browne's view, to secure State Registration.

In the alternative all trained nurses, who place first the best interests of their profession and the present necessity to secure State Registration with dispatch, can put the position right and remove all cause for further delay by entering their names on the College Register and getting their friends to do likewise. If they do this at once the College Register will speedily contain upwards of 10,000 names, and victory should be assured.

We have reason to know that the Building and Endowment Funds of the College, and also the Tribute Fund, thanks to the indomitable energy of the British Women's Hospital Committee, are bound to be forthcoming without doubt. It will be grate-

**ROUND THE HOSPITALS—(*continued*).**

ful to the Nation if trained nurses now wake up to a realisation of the true position of matters in regard to the College and their own best interests. Nurses might then, with keen enjoyment, commence a neck-and-neck race with the British Women's Hospital Committee, to see which of them can first accomplish, completely, the object which each has in hand, and both desire completed. Will Endowment and Tribute precede Registration, or *vice versâ*? In these days every working woman is coming to realise the truth, which is that God helps those who help themselves. It is a world of give and take, and where there is more than one interest concerned, the necessary way to success is for all parties to unite, and work with a will to secure the best results for each and all.

Under the auspices of the British Women's Hospital Committee a concert in aid of the Nation's Fund for Nurses was given on the 8th instant at Queen's Hall by the Strolling Players' Amateur Orchestral Society. There was a large and appreciative audience. Mr. Joseph Ivimey was the conductor, and his new *Suite de Ballet* entitled "Nursery Rhymes," played with a great deal of piquant expression by his orchestra, was one of the most attractive items on the programme. Mr. Albert Sammons played the violin with excellent feeling and technique, and gave several of his well-known solos in his very best manner. Miss Marguerite Nielka sang the Page's Song from "Les Huguenots" with dramatic force. Miss Nielka also sang an English, a Russian, and a French song, and her beautiful voice thrilled the audience, and gave them infinite pleasure. The grateful thanks of British nurses, and all interested in the Nation's Fund for Nurses —and who is not?—are due to all who contributed to the success of this concert.

A special general meeting of the members of the R.B.N.A. was held at 11 Chandos Street, W., on the 12th instant, Dr. Kenneth Stewart in the chair. The meeting was well attended. Mr. Paterson explained at great length the happenings since the July meeting and his responsibility in the matter. Mr. Comyns Berkeley, in resigning the treasurership, made an explanatory statement. The meeting approved the course taken in regard to the draft Supplemental Charter, and the non-completion of the agreement entered into with the College of Nursing, Limited.

Colonel W. L. W. Marshall, C.M.G., commanding the Huddersfield War Hospital, recently announced a gift which will make the hospital absolutely complete. The donors are Mr. Jesse Lumb and Mr. William Lumb, of Eldon House, Almondbury, members of a well-known firm of West-Riding worsted-spinners, and their gift takes the form of two cheques for £250 each, for the purpose of erecting a small isolation block at the hospital for use as sick quarters for the nurses. In only too many hospitals no adequate provision is made for sick nurses, and if one falls ill she has to be "warded" at once, even though the malady may be slight. When a nurse is attacked with what may be taken to be an infectious disease, the difficulty of looking after her, until the doubt is settled, is often very great. We are pleased to record an instance where this much-needed provision has been made generously.

The patient in the next bed to the quiet man, but a humorist, had been ordered to have stout. When the surgeon, on his morning round, inquired of the humorist how he felt, he said: "I don't feel very well, sir. I had a horrible dream last night." "That will be all right," said the surgeon. "Battle-fighting again in your sleep, I expect. You'll soon get out of that habit." "It wasn't battle-fighting at all, sir," said a pathetic voice, "the fact is, I dreamt there was a bottle of stout beside my bed, and just when I thought I was going to have some, I put out my hand and found there wasn't any there."

**☛ It is our invariable practice to pay for all items of news which we may publish. The minimum payment is 5s. Paragraphs of about twenty lines in length—that is to say, of 150 words or less—are preferred. Contributions should be addressed to The Editor, The Hospital, 28 and 29 Southampton Street, Strand, London, W.C. 2, and, to be in time for the current week's issue, should reach him by the first post on Mondays.**

**Handicapped Uniforms.**

The Starch Shortage (Lance-Cpl. J. H. Dowd).

For Nursing Affairs in Ireland, see p. 230.

## Figures and Tables

**Figure f1:**
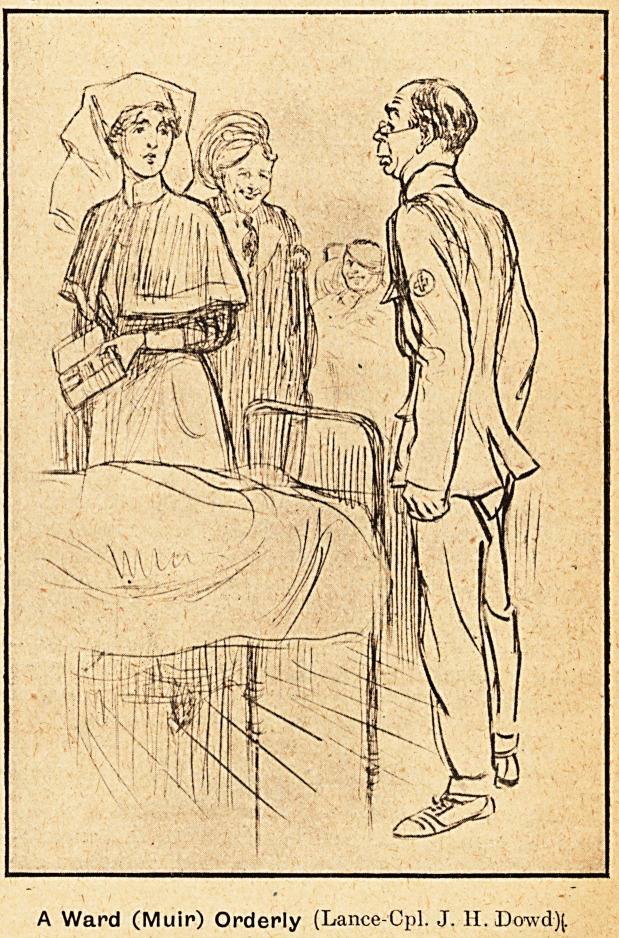


**Figure f2:**